# Functional Analysis of Nuclear Factor Y in the Wing-Dimorphic Brown Planthopper, *Nilaparvata lugens* (Hemiptera: Delphacidae)

**DOI:** 10.3389/fgene.2020.585320

**Published:** 2020-11-03

**Authors:** Hao-Hao Chen, Yi-Lai Liu, Xin-Yang Liu, Jin-Li Zhang, Hai-Jun Xu

**Affiliations:** ^1^Institute of Insect Sciences, Zhejiang University, Hangzhou, China; ^2^Ministry of Agriculture Key Laboratory of Molecular Biology of Crop Pathogens and Insect Pests, Zhejiang University, Hangzhou, China; ^3^State Key Laboratory of Rice Biology, Zhejiang University, Hangzhou, China

**Keywords:** *Nilaparvata lugens*, nuclear factor Y, wing development, ecdysone, reproduction

## Abstract

Nuclear factor Y (NF-Y) is a heterotrimeric transcription factor with the ability to bind to a CCAAT box in nearly all eukaryotes. However, the function of NF-Y in the life-history traits of insects is unclear. Here, we identified three NF-Y subunits, *Nl*NF-YA, *Nl*NF-YB, and *Nl*NF-YC, in the wing-dimorphic brown planthopper (BPH), *Nilaparvata lugens*. Spatio-temporal analysis indicated that *NlNF-YA*, *NlNF-YB*, and *NlNF-YC* distributed extensively in various body parts of fourth-instar nymphs, and were highly expressed at the egg stage. RNA interference (RNAi)-mediated silencing showed that knockdown of *NlNF-YA*, *NlNF-YB*, or *NlNF-YC* in third-instar nymphs significantly extended the fifth-instar duration, and decreased nymph-adult molting rate. The addition of 20-hydroxyecdysone could specifically rescue the defect in adult molting caused by *NlNF-YA*^RNAi^, indicating that *Nl*NF-Y might modulate the ecdysone signaling pathway in the BPH. In addition, *NlNF-YA*^RNAi^, *NlNF-YB*^RNAi^, or *NlNF-YC*^RNAi^ led to small and moderately malformed forewings and hindwings, and impaired the normal assembly of indirect flight muscles. Adult BPHs treated with *NlNF-YA*^RNAi^, *NlNF-YB*^RNAi^, or *NlNF-YC*^RNAi^ produced fewer eggs, and eggs laid by these BPHs had arrested embryogenesis. These findings deepen our understanding of NF-Y function in hemipteran insects.

## Introduction

Nuclear transcription factor Y (NF-Y) is an evolutionarily conserved transcription factor that exists in almost all organisms, from prokaryotes to eukaryotes (Dorn et al., 1987; Laloum et al., 2013). NF-Y exerts differential regulation on a wide variety of genes through binding to a CCAAT box, one of the most ubiquitous elements in eukaryotic promoters (Li et al., 1992; Mantovani, 1998; Suzuki et al., 2001). NF-Y consists of three subunits, NF-YA, NF-YB, and NF-YC, all required for DNA-binding (McNabb et al., 1995; Sinha et al., 1995; Bi et al., 1997; Mantovani, 1999). Accumulated evidence indicates that animal NF-Y is essential for numerous biological processes involved in proliferation and apoptosis, cancer and tumorigenesis, stress responses, growth, and development (Li et al., 2018). For instance, the deletion of the mouse *NF-YA* homolog causes early embryo lethality (Bhattacharya et al., 2003). Down-regulation of mouse *NF-YA* was found to reduce the expression of several cell cycle control genes in differentiated muscle cells, suggesting NF-YA is involved in specifying myogenic fate (Gurtner et al., 2003). Knockdown or overexpression of the *Drosophila melanogaster NF-YA* homolog in all tissues results in lethality at the larval stage, indicating that a certain level of NF-YA is necessary for viability in flies (Yoshioka et al., 2007). However, he contribution of NF-Y homologs to the life-history traits of other insect species has yet to be reported.

The wing-dimorphic brown planthopper (BPH), *Nilaparvata lugens* (Hemiptera: Delphacidae), is the most destructive pest of rice in Asia (Xue et al., 2014), causing a loss in rice production of more than $300 million annually in Asia. It feeds exclusively on the phloem sap of the rice plant and transmits plant viruses such as rice ragged stunt virus and rice grassy stunt virus, resulting in loss of plant vigor and reduced yield (Backus et al., 2005; Otuka, 2013). The BPH is a hemimetabolous insect, the newly hatched first-instar nymphs look like miniature adults except for the absence of wings and sexual immaturity, and grow gradually within increasing stages. The BPH has five nymphal stages, and wing buds grow with each of these stages, but short- and long-winged morphs are externally indistinguishable until the adults emerge (Xu and Zhang, 2017). Long-winged BPH adults have well-developed forewings, hindwings, and indirect flight muscles (IFM), whereas, short-winged adults have undeveloped forewings and rudimentary hindwings (Xu and Zhang, 2017; Zhang et al., 2019). Wing dimorph and high fecundity are the two most important biological features contributing to the ecological success of the BPH (Zhang et al., 2019). Recently, facilitated by the development of genetic tools (Xu et al., 2013; Xue et al., 2018) and genomic information (Xue et al., 2014), the molecular basis underlying wing dimorphism (Xu et al., 2015) and population resurgence (Wu et al., 2020) are emerging, which offers a basis for the design of new control agents to combat BPH infestation.

In the present study, we identified the BPH *NF-YA* (*NlNF-YA*), *NF-YB* (*NlNF-YB*), and *NF-YC* (*NlNF-YC*) homologs by searching the BPH genomic and transcriptomic databases. RNA interference (RNAi)-mediated gene silencing showed that *Nl*NF-YA, *Nl*NF-YB, and *Nl*NF-YC all played pivotal roles in nymph growth, wing formation, IFM development, and reproduction. In addition, we found that NF-Y regulated adult eclosion most likely through linking to the ecdysone signaling pathway. Our findings deepen our understanding of the function of NF-Y in hemipteran insects.

## Materials and Methods

### Insects

The BPH strain was originally collected from a rice field in Hangzhou, China. Insects were reared on rice seedlings (rice variety: Xiushui 134) in a walk-in chamber at 26 ± 0.5°C, with a light: dark photoperiod of 16: 8 h and relative humidity of 50 ± 5%.

### Gene Identification and Sequence Analysis

The amino acid sequences of *Drosophila* NF-Y homologs were used to screen BPH genomic and transcriptomic databases for homologs. Total RNA was isolated from BPH nymphs using RNAiso plus (Takara #9109) according to the manufacturer’s protocol. For cDNA syntheses, 450 ng of total RNA was reversely transcribed in a 10 μL reaction with HiScript QRT SuperMix (Vazyme #R223-01). The *NlNF-YA*, *NlNF-YB*, and *NlNF-YC* sequences were amplified from cDNA using fidelity DNA polymerase (Toyobo #930700) with NlNF-YA-F/R, NlNF-YB-F/R, and NlNF-YC-F/R primer pairs, respectively (Supplementary Table 1). The PCR product was cloned and the sequence was determined by Sanger sequencing.

To identify the exon-intron construction of *NlNF-Y*, open reading frames (ORFs) of *NlNF-YA*, *NlNF-YB*, and *NlNF-YC* were used to search a BPH genomic database (Xue et al., 2014). For phylogenetic analysis, 39 NF-Y sequences from 13 species including *D. melanogaster*, *N. lugens*, *Acyrthosiphon pisum*, *Apis cerana*, *Bemisia tabaci, Bombyx mori*, *Cryptotermes secundus*, *Halyomorpha halys*, *Monomorium pharaonis*, *Nasonia vitripennis*, *Nicrophorus vespilloides*, *Tribolium castaneum*, and *Zootermopsis nevadensis* were used for sequence alignment. A phylogenetic tree was constructed with maximum-likelihood method and 1000 bootstraps using the MEGA 7 program (Kumar et al., 2016).

### Spatio-Temporal Expression Pattern of *NlNF-Y*

Total RNA was isolated from eggs (*n* = 100), first-instar (*n* = 100), second-instar (*n* = 50), third-instar (*n* = 50), fourth-instar (*n* = 30), fifth-instar nymphs (*n* = 15), and adult females (*n* = 15), which were laid or ecdysed within 24 h. To examine tissue distribution, we dissected the head (*n* = 50), antenna (*n* = 200), tergum (*n* = 50), leg (*n* = 100), fat body (*n* = 30), cuticle (*n* = 50), and gut (*n* = 100) from four-instar nymphs for RNA extraction. First-strand cDNA was synthesized from total RNA (450 ng) using HiScript QRT SuperMix (Vazyme #R223-01). The synthesized cDNAs were 10-fold diluted and used as templates for quantitative real-time PCR (qPCR). The qPCR primers for *NlNF-Y* (Supplementary Table 1) were designed using Primer-Blast.^1^ The qPCR was conducted on a CFX96TM real-time PCR detection system (Bio-Rad) using the following conditions: denaturation for 3 min at 95°C, followed by 40 cycles at 95°C for 10 s, and 60°C for 30 s. The ribosomal protein S11 gene (*rps11*) was used as the internal reference gene (Yuan et al., 2014). The 2^–ΔΔCt^ method (Ct represents the cycle threshold) was used to measure the relative expression level (Livak and Schmittgen, 2001). Three independent biological replicates with three technical replicates were conducted for each experiment.

### RNAi and Microinjection

The dsRNAs were synthesized using a T7 High Yield Transcription Kit (Vazyme #TR101-02) according to the manufacturer’s instructions with primers containing the T7 RNA polymerase promoter at both ends (Supplementary Table 1). A dsRNA injection was carried out as in our previous study (Xu et al., 2015). Briefly, fourth-instar nymphs were anesthetized with carbon dioxide for 10–15 s. Approximately 150 ng dsRNA was microinjected into the mesothorax using a FemtoJet microinjection system (Eppendorf). To investigate whether disruption of *NlNF-YA* affected ecdysone signaling activity, fifth-instar nymphs (*n* = 10 for each of three replicates) were collected to examine the expression levels of *NlE74A* and *NlE75B* by qPCR using corresponding primers (Supplementary Table 1). To investigate RNAi efficiency, BPHs (*n* = 5 for each of three replicates) at 24 h after adult eclosion were collected for qPCR assay.

### Forewing Size and Hind Tibiae Length

Fourth-instar nymphs (36–48 h after ecdysis, hAE) were microinjected with dsRNAs targeting *NlNF-YA* (ds*NlNF-YA*), *NlNF-YB* (ds*NlNF-YB*), *NlNF-YC* (ds*NlNF-YC*), or *GFP* (ds*GFP*). After adult eclosion (24 h), images of the forewings and hind tibiae were captured with a DFC320 digital camera attached to a Leica S8AP0 stereomicroscope using a LAS digital imaging system (v. 3.8). Digital images of forewings (*n* = 20) and hind tibias (*n* = 20) were collected for the measurement of forewing size and hind tibia length using ImageJ (v. 1.47).

### Microinjection With 20-hydroxyecdysone (20E)

Fourth-instar nymphs (36–48 hAE) were microinjected with corresponding dsRNAs targeting each gene, and then nymphs were maintained on fresh rice seedings until the fifth-instar stage (within 48 h). Fifth-instar nymphs were microinjected with 20E (1 mg/ml) or distilled water, and the adult eclosion rate was calculated.

### Transmission Electron Microscopy

Fourth-instar nymphs (36–48 hAE) were microinjected with dsRNAs targeting *NlNF-YA*, *NlNF-YB*, *NlNF-YC*, or *GFP*. The thoraxes were dissected from adult females (24 h after emergence) for Transmission Electron Microscopy (TEM) as in our previous study (Xue et al., 2013). Briefly, samples were fixed in 2.5% glutaraldehyde overnight at 4°C. After fixation, samples were post-fixed in 1% osmium tetroxide for 1.5 h. Then, samples were dehydrated in a standard ethanol/acetone series, infiltrated and embedded in Spurr medium, and then superthin sections were cut. The sections were stained with 5% uranyl acetate followed by Reynolds’ lead citrate solution and observed under a JEM-1230 transmission electron microscope (JEOL).

### Fecundity Assay

Newly emerged females and males (within 12 h after eclosion) were microinjected with ds*NF-YA*, ds-*NF-YB*, ds*NF-YC*, or ds*Gfp.* Then each female was allowed to match with two males in a glass tube. Insects were removed at 5 days after matching, and eggs were counted under a Leica S8AP0 stereomicroscope.

### Data Analysis

Statistical analyses were performed using GraphPad Prism 7.0 (GraphPad Software). Means were compared using a two-tailed Student’s *t*-test and log-rank (Mantel-Cox) test at a significance level set at ^∗^*P* < 0.05, ^∗∗^*P* ≤ 0.01, and ^∗∗∗^*P* ≤ 0.001.

## Results

### *NlNF-YA*, *NlNF-YB*, and *NlNF-YC* Sequence Analysis

**FIGURE 1:**
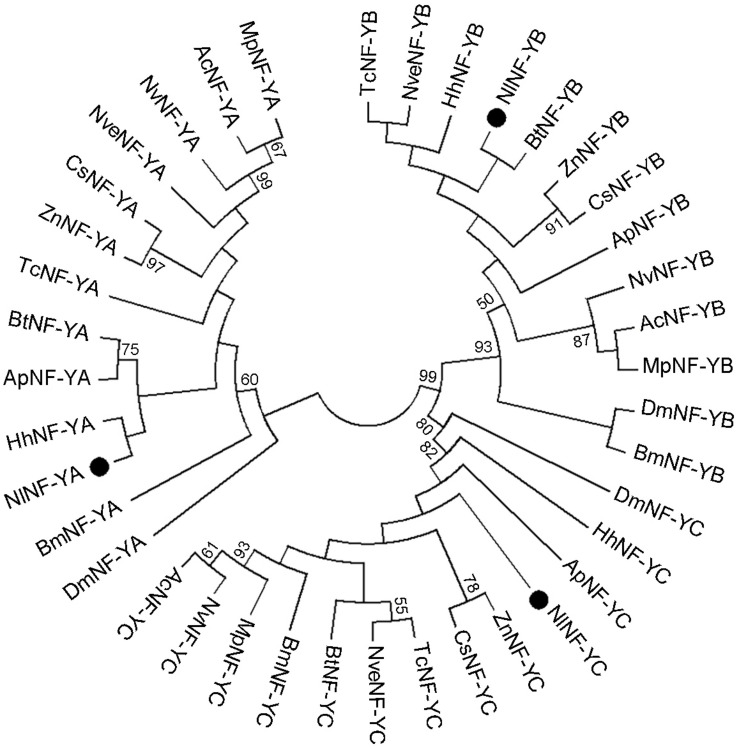
Phylogenetic analysis of *Nl*NF-Y members. The phylogenetic tree was constructed with 39 full-length *Nl*NF-Y members from 13 species using the maximum-likelihood method and bootstrapping was set to 1,000 replications. *Nl*NF-YA, *Nl*NF-YB, and *Nl*NF-YC are indicated by dots. Nl, *Nilaparvata lugens*; Dm, *Drosophila melanogaste*r; Ap, *Acyrthosiphon pisum*; Ac, *Apis cerana*; Bt, *Bemisia tabaci*; Bm, *Bombyx mori*; Cs, *Cryptotermes secundus*; Hh, *Halyomorpha halys*; Mp, *Monomorium pharaonis*; Nv, *Nasonia vitripennis*; Nve, *Nicrophorus vespilloides*; Tc, *Tribolium castaneum*, and Zn, *Zootermopsis nevadensis*.

We identified three genes encoding BPH NF-Y homologs, *NlNF-YA*, *NlNF-YB*, and *NlNF-YC*, in a BLAST search against BPH genomic (Xue et al., 2014) and transcriptome databases using the *D. melanogaster* NF-Y proteins as query sequences. The ORFs of *NlNF-YA*, *NlNF-YB*, and *NlNF-YC* were 1002-, 609-, and 1011-bp in length, encoding 333, 202, and 336 amino acid residues, respectively (Supplementary Figures 1–3). Exon-intron construction analysis showed that the *NlNF-YA*, *NlNF-YB*, and *NlNF-YC* ORFs consist of eight, five, and eight exons, located in scaffolds 6450/1657, 3414, and 1809, respectively. A phylogenetic analysis based on NF-Y homologs from 13 species showed that *Nl*NF-YA, *Nl*NF-YB, and *Nl*NF-YC together with their counterparts formed three distinct clusters (Figure 1), indicating that the BPH *NF-Y* genes we identified were authentic *NF-Y* homologs.

### Spatio-Temporal Analysis of *NlNF-YA*, *NlNF-YB*, and *NlNF-YC*

**FIGURE 2:**
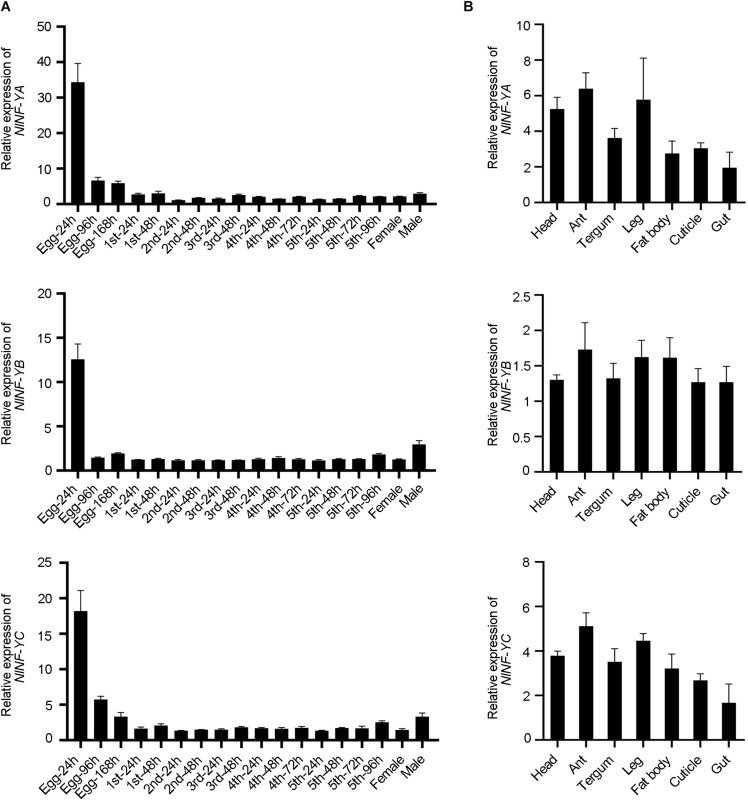
Spatio-temporal expression of *NlNF-Y* genes. **(A)** Developmental profile of *NlNF-YA*, *NlNF-YB*, and *NlNF-YC*. Total RNAs were isolated from eggs (*n* = 100), first-instar (*n* = 100), second-instar (*n* = 50), third-instar (*n* = 50), fourth-instar (*n* = 30), fifth-instar nymphs (*n* = 15), and adult females (*n* = 15). **(B)** Tissue distribution of *Nl*NF-Y. Total RNAs were extracted from the head (*n* = 30), antenna (ant, *n* = 200), tergum (*n* = 50), leg (*n* = 100), fat body (*n* = 30), cuticle (*n* = 50), and gut (*n* = 100) from fourth-instar nymphs. First-strand cDNA was synthesized using random primers, and qPCR was conducted using specific primers corresponding to each gene. The relative expression level was normalized by the ribosomal S11 gene (*rps11*). Bars represent s.d. derived from three independent biological replicates.

Spatio-temporal expression of *NlNF-YA*, *NlNF-YB*, and *NlNF-YC* was examined by qPCR. *NlNF-YA*, *NlNF-YB*, and *NlNF-YC* transcripts were readily detected from egg to adult stages, and relatively high levels were detected in eggs laid within 24 h (Egg-24 h) (Figure 2A), indicating that they might play important functions in egg development. Tissue distribution analysis showed that *NlNF-YA*, *NlNF-YB*, and *NlNF-YC* were evenly expressed in head, antenna, tergum, leg, fat body, cuticle, and gut of fourth-instar nymphs (Figure 2B), indicating *NlNF-Y* genes might be important for BPH growth and development.

### Knockdown of *NlNF-Y* Genes Leads to Small and Malformed Wings

**FIGURE 3:**
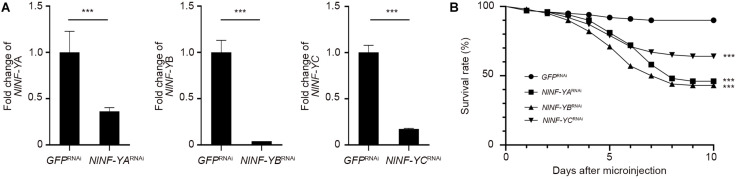
The survival rate of BPHs after dsRNA treatment. **(A)** Examination of RNAi efficiency. Fifth-instar nymphs (0–24 hAE) were microinjected with dsRNAs, and BPHs (*n* = 5 for each of three replicates) at 24 h after adult eclosion were collected for qPCR assay. The relative expression of *NlNF-YA*, *NlNF-YB*, and *NlNF-YC* was normalized to the expression of *rps11*. **(B)** The survival rate of BPHs after dsRNA treatment. Fourth-instar nymphs (*n* = 100) were microinjected with dsRNAs targeting *NlNF-YA*, *NlNF-YB*, *NlNF-YC*, or *Gfp*. Statistical analysis was performed by log-rank (Mantel-Cox) test. *NlNF-YA*^RNAi^, ds*NlNF-YB*^RNAi^, or ds*NlNF-YC*^RNAi^ significantly decreased the survivability of nymphs compared to *GFP*^RNAi^ (***P < 0.001).

**FIGURE 4:**
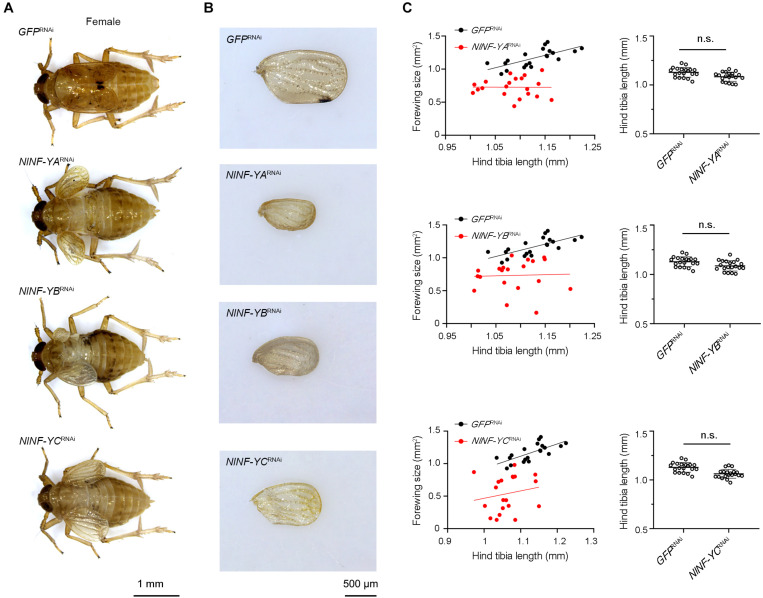
Knockdown of *NlNF-Y* reduces forewing size in short-winged BPHs. **(A)** Morphology of dsRNA-treated short-winged BPHs. **(B)** The size of forewings after dsRNA treatment. **(C)** Relative wing size and tibia length in BPHs with *NlNF-Y* or *Gfp* knockdown. Each dot represents the wing size and tibia length derived from an individual female (*n* = 20). Bars represent the mean ± s.d. derived from independent biological replicates (*n* = 20). Statistical comparisons between two groups were performed using a two-tailed Student’s *t*-test (n.s., non-significant).

To investigate whether *NlNF-YA*, *NlNF-YB*, and *NlNF-YC* played any roles in BPH development, fourth-instar short-wing-destined nymphs were microinjected with corresponding dsRNAs targeting each gene. At 48 h after microinjection, RNAi efficiency was examined by qPCR, which showed that dsRNA treatments significantly down-regulated the expression levels of *NlNF-YA*, *NlNF-YB*, and *NlNF-YC* by 63.7, 99.9, and 82.7%, respectively, relative to the *GFP*^RNAi^ treatment (Figure 3A). Notably, *NlNF-YA*^RNAi^, *NlNF-YB*^RNAi^, and *NlNF-YC*^RNAi^ caused high mortality, leading to approximately 60, 60, and 40% of nymphs died before adult eclosion (Figure 3B), respectively. The remaining nymphs could successfully molt into adults. However, these adults had moderately smaller and slightly malformed forewings relative to *GFP*^RNAi^ BPHs (Figures 4A,B). *NlNF-YA*^RNAi^, *NlNF-YB*^RNAi^, and *NlNF-YC*^RNAi^ specifically reduced forewing size but had marginal roles on hind tibiae length (Figure 4C). These data indicated that *NlNF-YA*, *NlNF-YB*, and *NlNF-YC* were essential for normal wing growth in BPHs.

### Knockdown of *NlNF-Y* Genes Leads to Defective IFM

**FIGURE 5:**
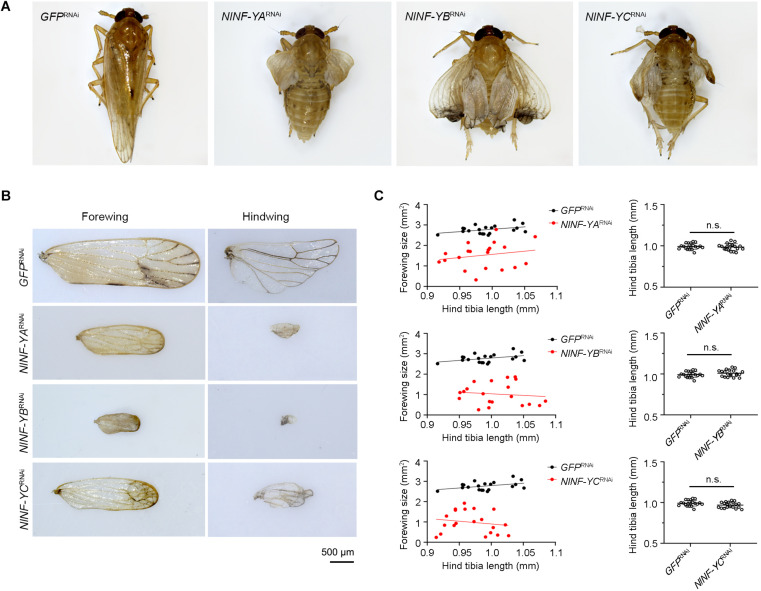
Knockdown of *NlNF-Y* reduces forewing size in long-winged BPHs. **(A)** Morphology of dsRNA-treated long-winged BPHs. **(B)** The size of forewings and hindwings after dsRNA treatment. **(C)** Relative wing size and tibia length in BPHs with *NlNF-Y* or *Gfp* knockdown. Each dot represents the wing size and tibia length derived from an individual female (*n* = 20). Bar represents mean ± s.d. derived from independent biological replicates (*n* = 20). Statistical comparisons between two groups were performed using a two-tailed Student’s *t*-test (n.s., non-significant).

**FIGURE 6:**
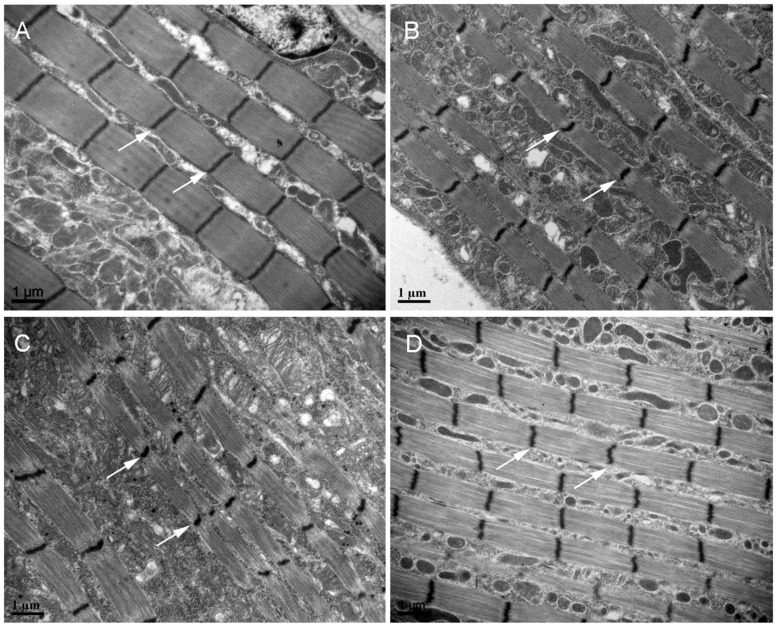
TEM of the IFM of dsRNA-treated long-winged BPHs. Fourth-instar nymphs were treated with dsRNAs targeting Gfp **(A)**, NlNF-YA **(B)**, NlNF-YB **(C)**, or NlNF-YC **(D)**. Thorax was dissected from female adults (24 h after eclosion) for TEM. The Z discs are indicated by arrows.

Because short-wing BPHs lack hindwings and IFM, we used long-wing BPHs to investigate whether *NlNF-YA*, *NlNF-YB*, and *NlNF-YC* had any effect on IFM development. For this, fourth-instar long-wing-destined nymphs were microinjected with corresponding dsRNAs targeting each gene. *NlNF-YA*^RNAi^, *NlNF-YB*^RNAi^, and *NlNF-YC*^RNAi^ gave rise to adults with curved forewings and hindwings (Figure 5A). In line with the phenotype in short-winged BPHs, smaller forewings and hindwings were observed in adults previously treated with either *NlNF-YA*^RNAi^, *NlNF-YB*^RNAi^, or *NlNF-YC*^RNAi^ (Figure 4B), although there was no discernable effect on hind tibia length (Figure 4C). Next, these adults were collected for IFM ultrastructure examination using TEM. In *GFP*^RNAi^ adults, sarcomeres were clearly divided by Z discs and well-organized (Figure 6). In contrast, *NlNF-YA*^RNAi^, *NlNF-YB*^RNAi^, or *NlNF-YC*^RNAi^ led to defective sarcomeres, with deformed Z discs and weakly organized myofibrillar. These events indicated that the *Nl*NF-Y complex might be essential for IFM assembly in BPH.

### Knockdown of *NlNF-Y* Genes Impairs Nymphal Growth and Adult Molting

**FIGURE 7:**
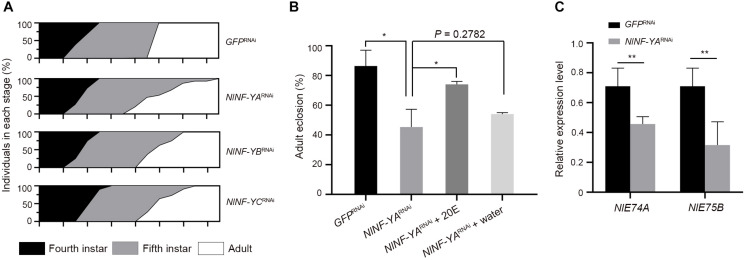
Nymphal duration and adult eclosion of dsRNA-treated BPHs. **(A)** Third-instar nymphs (0–24 hAE) were microinjected with dsRNAs targeting *NlNF-YA* (*n* = 660), *NlNF-YB* (*n* = 435), *NlNF-YC* (*n* = 480), or *Gfp* (*n* = 442). The curve in different developmental stages indicates the molting ratio. (**B**) The nymph-adult eclosion rate of BPHs. Fourth-instar nymphs (36–48 hAE) were microinjected with dsRNAs targeting *NlNF-YA* or *Gfp*, and then 20E or distilled water was microinjected when nymphs proceeded the fifth-instar stage. **(C)** The expression of *NlE74A* and *NlE75B* in context of *NlNF-YA* knockdown. The relative expressions of *NlE74A* and *NlE75B* were normalized to the expression of *rps11*. Statistical comparisons between two groups were performed using two-tailed Student’s *t*-test (*P < 0.05, and **P < 0.01).

Given that *NlNF-Y* genes were evenly expressed across nymphal stages (Figure 2A), we investigated whether knockdown of *NlNF-Y* genes would affect nymph growth. For this purpose, third-instar nymphs were microinjected with corresponding dsRNAs targeting each gene. Nymphs treated with *NlNF-YA*^RNAi^, *NlNF-YB*^RNAi^, or *NlNF-YC*^RNAi^ had an extended fifth-instar duration compared with those treated with *GFP*^RNAi^ (Figure 7A), with *NlNF-YA*^RNAi^ showing the most profound effect (Figure 7A). In addition, the majority of *NlNF-YA*^RNAi^-treated nymphs died before adult emergence (Figure 7B), indicating that the disruption of the NF-Y complex impaired nymph-to-adult ecdysis.

Given that ecdysone is a hormone that initiates all major developmental transitions from the egg, to larva, to pupa, to adult in insects (Gilbert et al., 2002; Dubrovsky, 2005), we asked whether it mediated the effects of *Nl*NF-YA on adult molting. For this purpose, we first assessed the expression levels of two early ecdysone response genes, *NlE74A* and *NlE75E* (Dubrovsky, 2005), in the context of *NlNF-YA* knockdown. *NlNF-YA*^RNAi^ significantly reduced both *NlE74A* and *NlE75E* transcripts compared with *GFP*^RNAi^ (Figure 7C), indicating that knockdown of *NlNF-YA* might impair ecdyson signaling activity. Following this observation, we asked whether the addition of ecdysone could rescue the *NlNF-YA*^RNAi^ defect. To this end, fourth-instar nymphs were microinjected with ds*NlNF-YA*, followed by microinjection with 20E when nymphs proceeded to the fifth-instar at 48 h after eclosion. The addition of 20E could partially restore the molting defect caused by *NlNF-YA*^RNAi^ (Figure 7B) compared with the addition of water, indicating that *NlNF-YA*^RNAi^ decreased adult eclosion at least partially through the ecdysone signaling pathway.

### Knockdown of *NlNF-Y* Genes Impairs Reproduction

**FIGURE 8:**
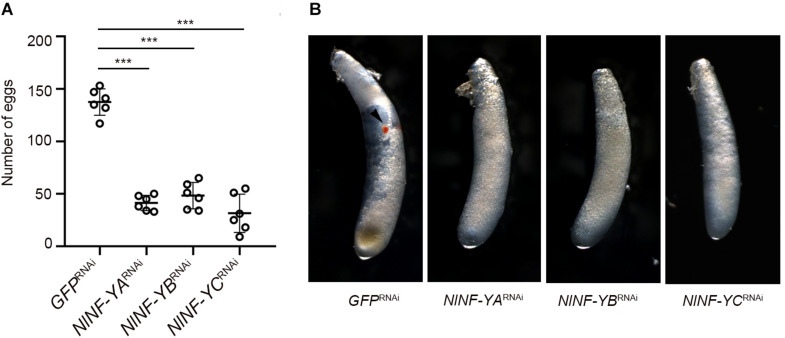
Fecundity of adults with gene knockdown and phenotype of eggs produced. **(A)** Newly emerged adults (0–12 hAE) were treated with dsRNAs targeting each gene. The dsRNA-treated females and males were allowed to mate and laid eggs for 5 days. Each circle represents eggs produced by an individual female (*n* = 6). Bars represent the mean ± s.d. Statistical comparisons between two groups were performed using a two-tailed Student’s *t*-test (***P < 0.001). **(B)** Morphology of eggs deposited after 5 days by dsRNA-treated BPHs. The eye pigmentation is indicated by an arrow head.

Given that relatively high amounts of *NlNF-YA*, *NlNF-YB*, and *NlNF-YC* transcripts were detected at the early egg stage (Figure 2A), we asked whether *NlNF-YA, NlNF-YB*, and *NlNF-YC* contributed to BPH reproduction. Female and male adults at 12 h after eclosion were microinjected with corresponding dsRNAs targeting each gene, and then allowed to mate and to lay eggs for 5 days. BPHs treated with *NlNF-YA*^RNAi^, *NlNF-YB*^RNAi^, and *NlNF-YC*^RNAi^ laid substantially fewer eggs than those treated with *GFP*^RNAi^ (Figure 8A). In addition, the eggs laid by BPHs previously treated with *NlNF-YA*^RNAi^, *NlNF-YB*^RNAi^, or *NlNF-YC*^RNAi^ failed to develop eye pigmentation, a characteristic hallmark of egg development, indicating *NlNF-Y* genes might be essential for embryogenesis (Figure 8B).

## Discussion

In this study, we investigated the functions of individual *NlNF-Y* members in the BPH during nymphal development, adult molting, wing growth, IFM development, and reproduction. The results demonstrated that *Nl*NF-Y played pivotal roles in these processes. Depletion of *NlNF-YA*, *NlNF-YB*, and *NlNF-YC* resulted in extended nymphal duration, affected nymph-adult molting progress, reduced wing size, disrupted IFM assembly, reduced egg production, and impaired egg development. These findings provide an impetus to understand the function of NF-Y in life-history traits of insects.

The CCAAT box is one of the most common cis-acting elements found in the promoter and enhancer regions of various genes in eukaryotes (Li et al., 1992). An analysis of a large database of 1,031 human promoters indicated that the CCAAT box is present in 63% of them (Suzuki et al., 2001). NF-Y is the major CCAAT box recognizing protein that binds to DNA with high specificity and affinity (Bi et al., 1997; Mantovani, 1998). Knockdown or overexpression of the *Drosophila NF-YA* homolog with different GAL4 also drives lethality in the *Drosophila* pharate adult stage, possibly by influencing disc specification (Yoshioka et al., 2007; Ly et al., 2013). A similar phenomenon was observed in BPH, depleting *NlNF-YA*, *NlNF-YB*, and *NlNF-YC* delayed fifth-instar development and led to high lethality, indicating that the CCAAT box might also be a common cis-acting element in the BPH. In addition, we noticed that depletion either *NlNF-YA*, *NlNF-YB*, or *NlNF-YC* led to small and malformed forewings and hindwings. As a wing-dimorphic insect, fifth-instar BPH nymphs have the ability to develop into either short-winged or long-winged adults. A recent finding showed that cells of short wing pads are largely in the G2/M phase of the cell cycle, whereas those of long wings are largely in G1, indicating that cell cycle progression is necessary for wing morph determination (Lin et al., 2020). These observations are in line with previous reports that cell cycle-related genes are the main target genes of NF-Y (Zwicker et al., 1995; Bolognese et al., 1999; Farina et al., 1999; Kao et al., 1999; Korner et al., 2001; Manni et al., 2001). In addition, the depletion of *NlNF-Y* not only affected wing size, but also led to an IFM defect, the latter tissue is only present in long-winged adults. Based on these events, we speculate that NF-Y might be tightly involved in regulating alternative wing morphs in the BPH although this needs to be further confirmed experimentally.

It is of interest that the depletion of *NlNF-YA* significantly decreased the adult molting rate, and this defect could be partially rescued by the addition of 20E. In addition, the depletion of *Nl*NF-YA significantly reduced the expression levels of *NlE74A* and *NlE75B*, the downstream genes of the ecdysone pathway. These findings indicated that *Nl*NF-Y might affect the ecdysone pathway although the underlying mechanism remains unknown. Although there remains much to be done, our findings provided a first glimpse of the function of NF-Y in hemipteran insects.

## Data Availability Statement

The original contributions presented in the study are included in the article/Supplementary Material, further inquiries can be directed to the corresponding author.

## Author Contributions

H-HC and H-JX designed the experiment and wrote the manuscript. H-HC, Y-LL, X-YL, and J-LZ conducted the experiments. H-JX managed and directed the project. All authors contributed to the article and approved the submitted version.

## Conflict of Interest

The authors declare that the research was conducted in the absence of any commercial or financial relationships that could be construed as a potential conflict of interest.

## Footnotes

1 https://www.ncbi.nlm.nih.gov/tools/primer-blast/

## Supplementary Material

Supplementary Table 1 Primers used in this study.
